# Undetectable Changes in Image Resolution of Luminance-Contrast Gradients Affect Depth Perception

**DOI:** 10.3389/fpsyg.2016.00242

**Published:** 2016-02-23

**Authors:** Yoshiaki Tsushima, Kazuteru Komine, Yasuhito Sawahata, Toshiya Morita

**Affiliations:** ^1^Three-Dimensional Image Research Division, NHK Science and Technology Research LabsTokyo, Japan; ^2^Universal Communication Research Institute, National Institute of Information and Communications TechnologyKyoto, Japan; ^3^Sutokuin LabOsaka, Japan

**Keywords:** depth sensation, depth perception, display resolution, shading, conscious/unconscious

## Abstract

A great number of studies have suggested a variety of ways to get depth information from two dimensional images such as binocular disparity, shape-from-shading, size gradient/foreshortening, aerial perspective, and so on. Are there any other new factors affecting depth perception? A recent psychophysical study has investigated the correlation between image resolution and depth sensation of Cylinder images (A rectangle contains gradual luminance-contrast changes.). It was reported that higher resolution images facilitate depth perception. However, it is still not clear whether or not the finding generalizes to other kinds of visual stimuli, because there are more appropriate visual stimuli for exploration of depth perception of luminance-contrast changes, such as Gabor patch. Here, we further examined the relationship between image resolution and depth perception by conducting a series of psychophysical experiments with not only Cylinders but also Gabor patches having smoother luminance-contrast gradients. As a result, higher resolution images produced stronger depth sensation with both images. This finding suggests that image resolution affects depth perception of simple luminance-contrast differences (Gabor patch) as well as shape-from-shading (Cylinder). In addition, this phenomenon was found even when the resolution difference was undetectable. This indicates the existence of consciously available and unavailable information in our visual system. These findings further support the view that image resolution is a cue for depth perception that was previously ignored. It partially explains the unparalleled viewing experience of novel high resolution displays.

## Introduction

Obtaining depth information from a visual image on the retina, creating the third dimension from two dimensional images, is one of the most mysterious functions in our visual system. Considerable research has suggested a variety of ways to perceive depth, such as binocular disparity (Barlow et al., 1967; Pettigrew et al., 1968), shape-from-shading (Horn, 1975; Ramachandran, 1988), blur (Vishwanath, 2012), size gradient/foreshortening (Blake et al., 1993), or combination of these factors (Welchman et al., 2005). Are there any other new factors affecting depth perception?

**FIGURE 1:**
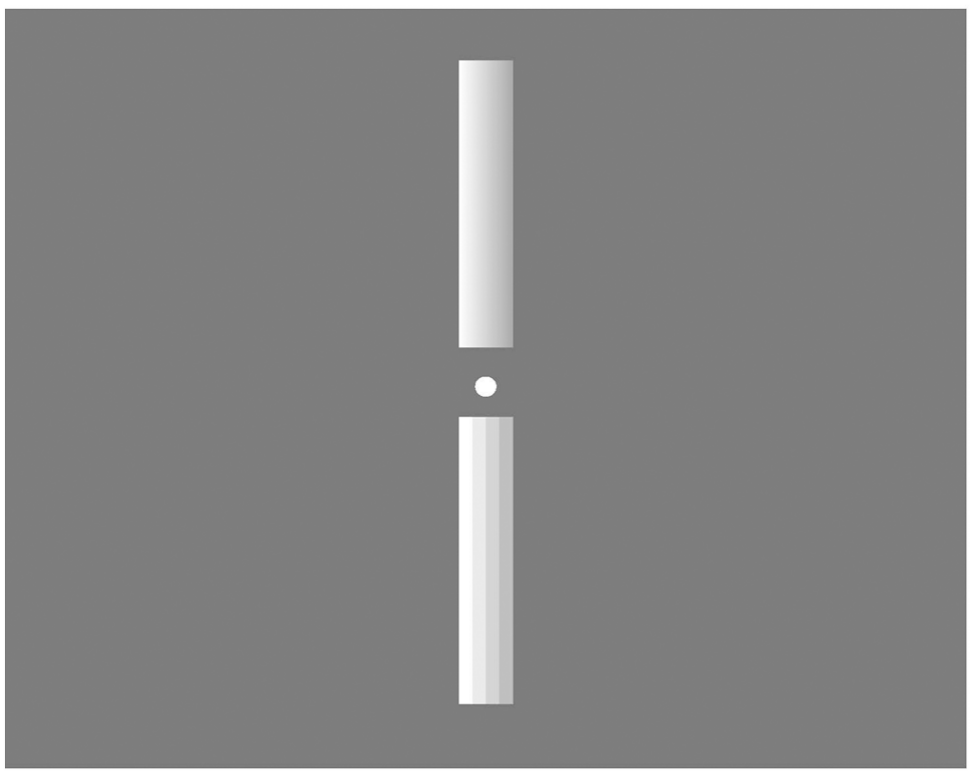
**Two different resolution rectangles containing gradual luminance-contrast changes (Tsushima et al., 2014).** Higher resolution (upper) and lower resolution (bottom).

Recently, Tsushima et al. (2014) examined the relationship between spatial resolution of a visual image and depth sensation, because lately developed high-resolution displays can give unprecedented sensation such as a sense of realness (Masaoka et al., 2013). They presented two different resolution rectangles that contained gradual luminance-contrast changes for defining the depth information (Ramachandran, 1988; O’Shea et al., 1994) (**Figure 1**), and asked participants to report which image gave rise to stronger depth sensation with monocular viewing (Depth task). Also, to test whether or not participants realized the resolution difference, they conducted another task with the same stimuli set, in which participants were asked to report which image had higher resolution with monocular viewing (Resolution task).

They found that the higher resolution image produced stronger depth sensation, and concluded that the image resolution greatly correlates with depth sensation. In addition, participants selected the higher resolution image with stronger depth sensation even when they did not notice the resolution difference. Since the appearance of luminance-contrast changes induces one of fundamental depth sensations (Ramachandran, 1988; Hou et al., 2006), using such cylinder image (**Figure 1**) was a good method for investigating the relationship between image resolution and depth perception. Moreover, unlike other studies, Tsushima et al. (2014) focused on image resolution, which is highly relevant for modern media technology.

However, such Cylinder images seem to lack the precision to psychophysically examine monocular depth perception of luminance-contrast differences, because it is not fitted to the receptive field aspect of neurons in primary visual cortex (Judson and Palmer, 1987). Actually, it might bring consequences that the participants did the task with focusing on the wrong image feature. For example, with Cylinder, there is a possibility that participants’ decision in the Depth task is greatly influenced by not the depth information but the aspect of the edge line between Cylinder and the background. This raised the question whether or not the previous findings may be generalized. To investigate the correlation between image resolution and depth perception of luminance-contrast difference/change more accurately, we should use more appropriate visual images having smoother and more edgeless luminance-contrast gradients, such as Gabor patch (Judson and Palmer, 1987).

To verify and generalize those previous findings, here we conducted similar psychophysical experiments with using Gabor patches (Experiment 1) and Cylinder (Experiment 2), and examined the relationship between image resolution and depth sensation. In Depth task, two different-resolution images were presented on a display, and participants were asked to report which image they perceived stronger depth with monocular viewing. In the same method as the previous study (Tsushima et al., 2014), in order to test whether or not participants can detect the resolution difference, we conducted Resolution task in which they were required to report which image had higher resolution, with the same visual stimulus set as Depth task.

## Materials and Methods

### Participants

In Experiment 1 (Gabor patch), 20 participants, aged from 20 to 39, normal or corrected vision, participated in a series of experiments. Ten participants were assigned for Depth task, and the other 10 participants for Resolution task.

In Experiment 2 (Cylinder), 16 participants, aged from 20 to 39, normal or corrected vision, participated in a series of experiments. Eight participants were assigned for Depth task, and the other eight participants for Resolution task.

All participants gave written informed consent, and the research was approved by the Ethics Committee of the NHK Science and Technology Research Laboratory, and in compliance with Declaration of Helsinki. Before starting the main experiment, we examined each participants’ visual acuity by using the tumbling *E* test with the same setting (equipment and environment) as the main experiment.

### Apparatus

A 27″ IPS-TFT color LCD Monitor (ColorEdge CG275W, EIZO Nanao Corp.) was used to present stimuli. The display had an area of 2560 × 1440 pixels with the pixel size of 0.2331 × 0.2331 mm^2^ and the contrast ratio of 850:1. Color calibration was performed before experiments to correct color balance and display gamma. We used 256 gray levels (8-bit color depth) to present the stimuli. Visual stimuli were presented by using Psychtoolbox 3 (http://psychtoolbox.org) on Windows 7. Viewing distance to the display was 3.21 m.

#### Measurement of the Actual Luminance

We used a photometer, Luminance Colorimeter (BM-7, Topcon Technohouse, Tokyo, Japan) and measured the luminance of each pixel component of the visual image used in the experiments for five times, then calculated the mean value for each point of observation.

### Stimuli

#### Experiment 1: Gabor Patch

**FIGURE 2:**
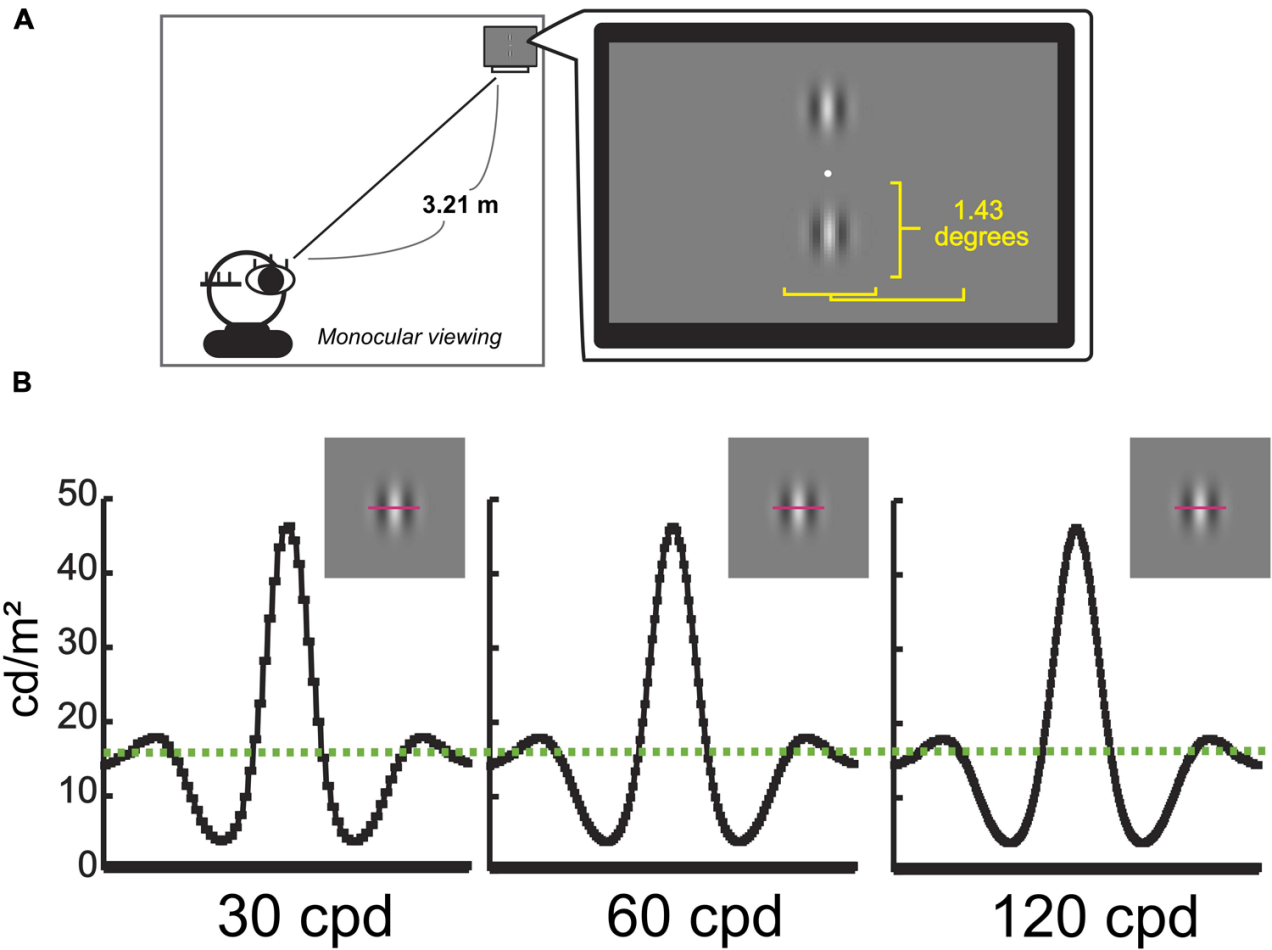
**Experimental environment for Gabor patch and example of Gabor patches.** **(A)** Environment for Gabor patch experiment. **(B)** Examples of Gabor patch in different resolutions, 30, 60, and 120 cpd. *Y*-axes represent the actual luminance value of each stimulus as a function of the horizontal spatial location indicating pink lines. A Green dashed line represents the actual luminance value of the background (13.9 cd/m^2^).

Two same-size but different-resolution Gabor patches were vertically presented on the computer display (**Figure 2A**). Since the results at the previous studies showed that the degree of depth sensation or realness sensation by changes of display resolution was saturated over 105 ∼155 cycle per degree (cpd) (Masaoka et al., 2013; Tsushima et al., 2014), we set the highest resolution as 120 cpd and presented three kinds of resolution images, 30, 60, and 120 cpd. The original Gabor patch was as follows: Spatial frequency of the Gabor was 3.5 cycles/degree, and the sigma of the Gaussian factor was 14°. The background was gray, 13.9 cd/m^2^. The maximum luminance of the Gabor was 46.3 cd/m^2^, and the minimum was 3.9 cd/m^2^ (**Figure 2B**). The processes to downconvert were as follows: A low-pass filter was applied to the image in spatial domain with the cut-off frequency of 0.5 (-6 dB at cut-off frequency) in normalized spatial frequency. The filtered image was re-sampled by a factor of 1/2 in both row-wise and column-wise, resulting in a half-size image to the original one. After that, each pixel of the image was replicated and interpolate among pixels to create same size but low-resolution images. These processes were applied to the images of 120 cpd (original image) to create the images of 60 cpd, and also applied to the images of 60 cpd to create the images of 30 cpd. In each trial, two kinds of three resolution stimuli were randomly chosen. In a complete experiment, there were four sessions, and each stimulus set was repeated 20 times in one session. So, the total experiment consisted of 3 combinations (of 2 images) × 20 repetitions × 4 sessions = 240 trials. The order of presentation of these conditions was randomly determined for each participant.

#### Experiment 2: Cylinder

**FIGURE 3:**
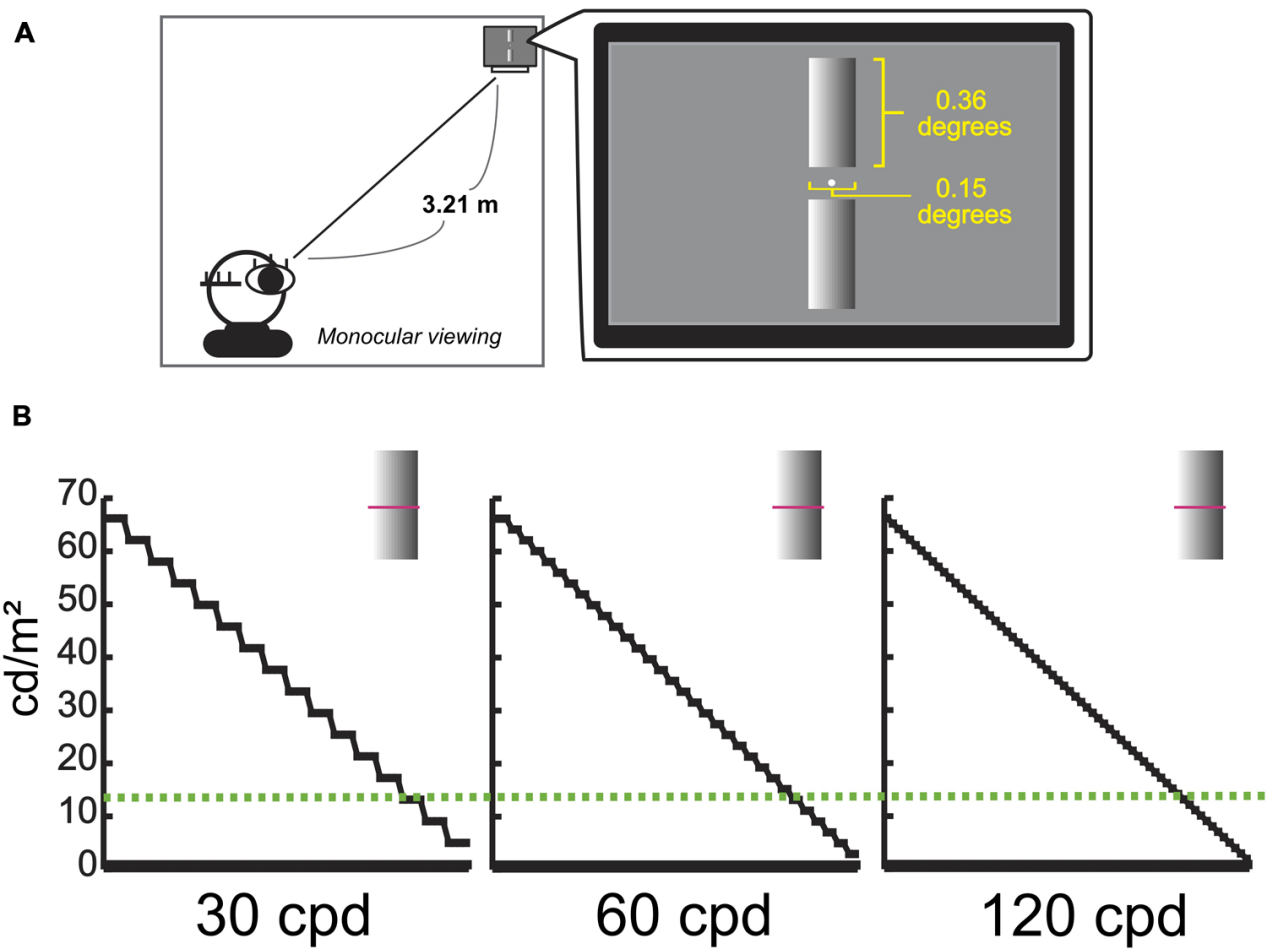
**Experimental environment for Cylinder and example of Cylinders.** **(A)** Environment for Cylinder experiment. **(B)** Examples of Cylinder in different resolutions, 30, 60, and 120 cpd. *Y*-axes represent the actual luminance value of each stimulus as a function of the horizontal spatial location indicating pink lines. A Green dashed line represents the actual luminance value of the background (13.9 cd/m^2^).

Participants were presented with two same-size but different-resolution cylinders on the computer display (**Figure 3A**), which had the gradual luminance-contrast change from one side to the other for providing depth information (Ramachandran, 1988; O’Shea et al., 1994). We presented three kinds of resolution images, 30, 60, and 120 cycle/degree (cpd). We set the highest resolution stimulus as the base image (the 120 cpd image consisting of 64 sub-bars, each sub-bar was equal in size; the right image at **Figure 3B**), and then it was down-converted using linear interpolation of the contrast, with fixing the mean luminance-contrast. Here, we denoted the number of sub-bar by *n*, and the contrast value of *i*-th sub-bar by *Ci* = 128 + (*n*/2 -*i*)^∗^(64/*n*). In each trial, two kinds of resolution images were randomly chosen within the stimuli set. In a complete experiment, there were four sessions, and each image set was repeated 32 times in one session. So, the total experiment consisted of 3 combinations (of 2 images) × 32 repetitions × 4 sessions = 384 trials. The order of presentation of these conditions was randomly determined for each participant.

### Procedure and Data analysis:

#### Depth Task

**FIGURE 4:**
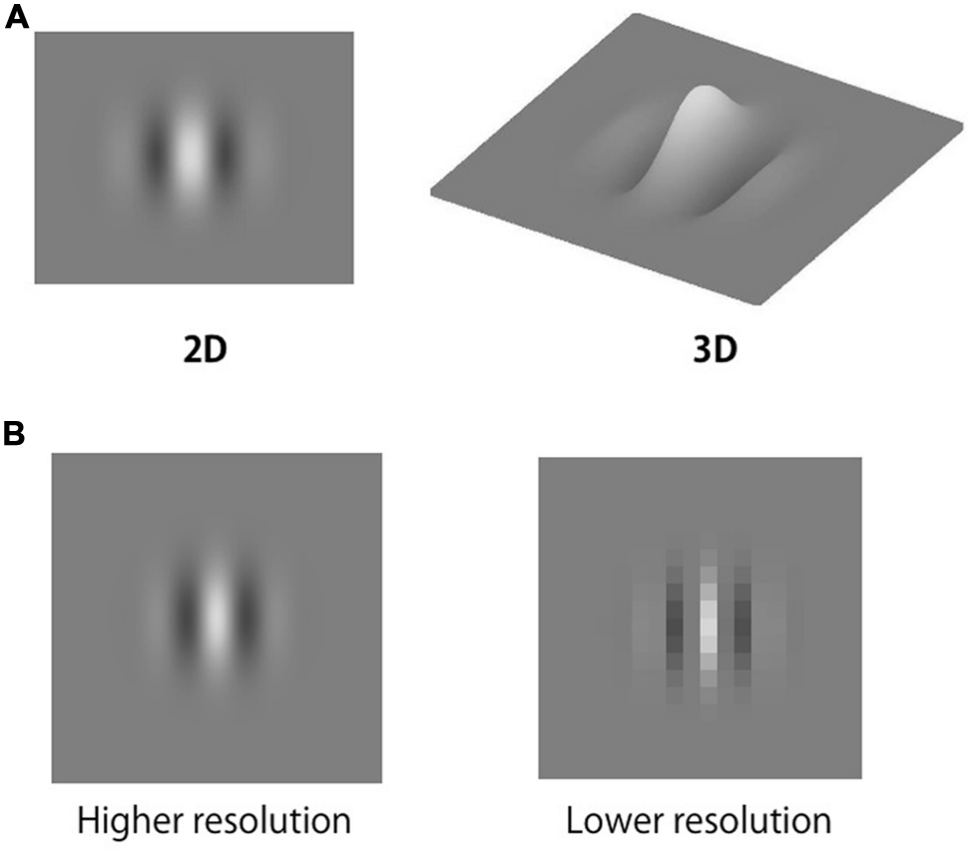
**Schematic illustrations for Depth and Resolution tasks.** **(A)** Schematic illustration for understanding the experimental idea, perceiving the depth sensation from Gabor patch. **(B)** Schematic illustration is provided for understanding the resolution.

Participants were instructed to report which image they perceived stronger depth with monocular viewing. Based on our preliminary studies, some naïve participant had difficulty of understanding the concept of getting the depth information from Gabor patch, therefore, we used the illustration and explained the experimental idea with **Figure 4A**.

In order to avoid that participants noticed the resolution difference between two stimuli, we did not tell them that there was resolution difference between two images. No feedback was given to the participants.

#### Resolution Task

To test whether or not the participants noticed the image resolution difference, we conducted the resolution task. It was identical to Depth task except that the participants were asked to report which image has higher resolution. In order to make participants to grasp the ideas of Resolution Task, we used the illustration and explained the experimental idea in Experiment 1 (**Figure 4B**).

To examine the relationship between depth sensation and image resolution, we used the method of paired comparison in which each image is matched one-on-one with each of the other image in our experiment. Thurstone-Mosteller (TM) model (Thurstone, 1927, 1994) (Case V) and Bradley-Terry (BT) model (Bradley, 1984) are well-known paired comparison model that can convert the paired comparison data to psychophysical scale rating. Since BT model is more mathematically developed (Handley, 2001) and produces more robust estimates of confidence intervals than Thurstone’s Case V model (Morovic, 2008), we used BT model to analyze the psychophysical data in this study. After finding the BT score (BTS), we calculated Pearson product-moment correlation coefficient between the BTS and the image resolutions to see the relationship between the image resolution and depth sensation, because the previous studies have shown an almost linear correlation from 30 to 120 cpd.

## Results

### Experiment 1: Gabor Patch

**FIGURE 5:**
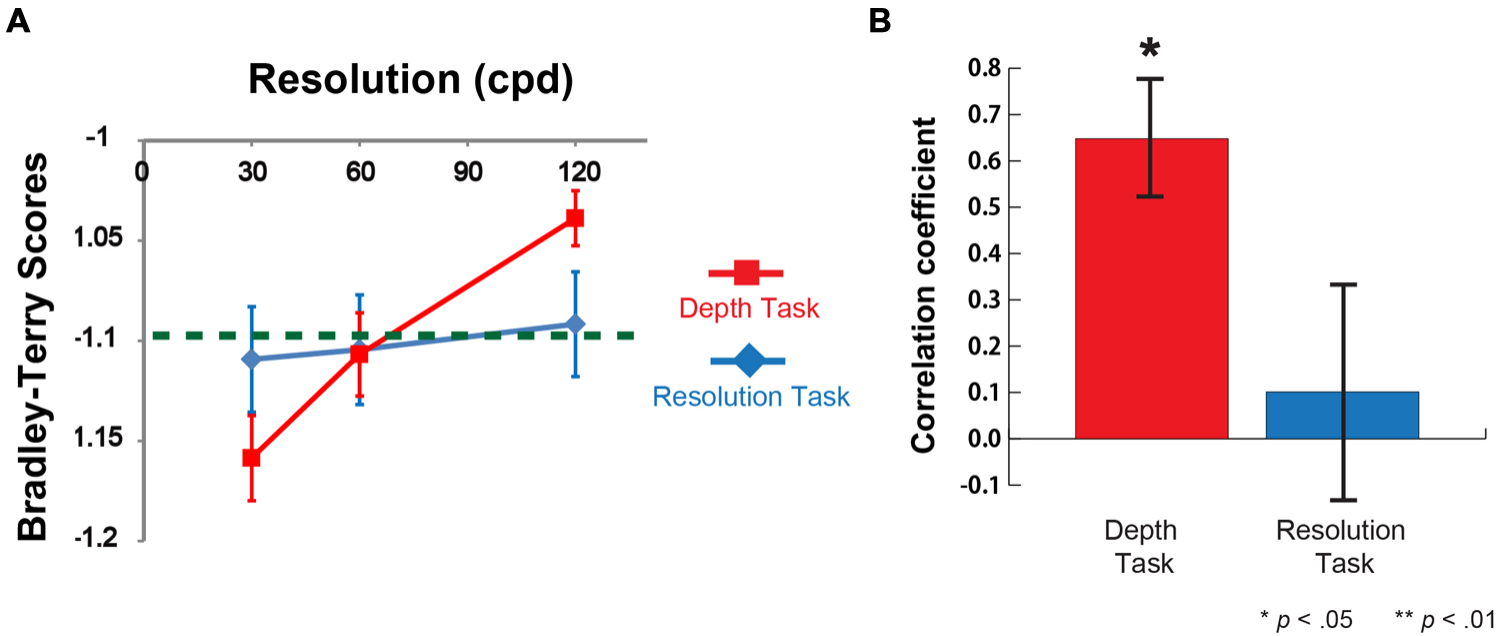
**Results of Depth task and Resolution task for Gabor patch.** **(A)** Mean Bradley-Terry score (BTS) for each image in the depth task (a red line) and the resolution task (a blue line) as a function of the resolution (cpd) (*n* = 10 for Depth Task and *n* = 10 for Resolution Task). Vertical error bars, ±1 SEM. A Green dashed line represents the chance-level choice rate (50% choice rate in two alternative forced choice). Mean BTS in the depth task at 120 cpd was significantly higher than chance-level (*t* = 4.37, df = 9, *p* < 0.01). Mean BTS in the depth task at 30 cpd was significantly lower than chance-level (*t* = -2.82, df = 9, *p* < 0.01) **(B)** Mean correlation coefficient between BTS and the image resolution in Depth and Resolution tasks (*n* = 10 each). The mean correlation coefficient between depth sensation and the image resolution was 0.65 (*n* = 10, *p* < 0.05, against zero). The mean correlation coefficient between resolution sensation and the image resolution was 0.10 (*n* = 10, *p* = 0.78, against zero).

The results show that participants’ depth sensation positively correlated with the stimulus resolution (a red line on **Figure 5A**, and the mean correlation coefficient between depth sensation and the image resolution was 0.65 ± 0.12 (*n* = 10, *p* < 0.05, against zero) on **Figure 5B**). On the other hands, in Resolution Task, there were no significant difference between the performance scores (a blue line) and the chance-level of choice rate (a green dashed line) (**Figure 5A**), and the mean correlation coefficient was 0.10 ± 0.21 (*n* = 10) (**Figure 5B**). This indicates that they were not able to discriminate the resolution difference. The results of the tumbling E visual acuity test show that there were no significant visual acuity differences between Depth and Resolution Task groups. Taken together, higher resolution image produces stronger depth sensation even without noticing the resolution difference.

### Experiment 2: Cylinder

**FIGURE 6:**
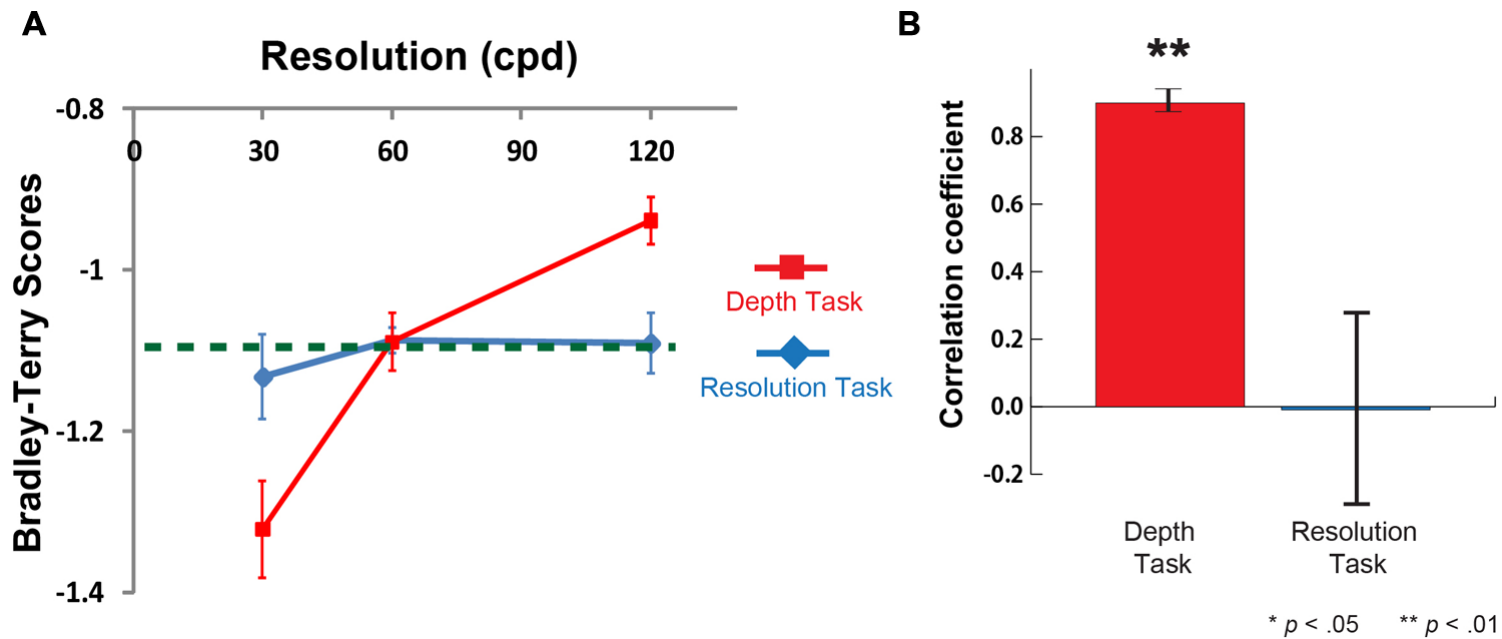
**Results of Depth task and Resolution task for Cylinder.** **(A)** Mean Bradley-Terry score for each image in the depth task (a red line) and the resolution task (a blue line) as a function of the resolution (cpd) (*n* = 8 for Depth Task and *n* = 8 for Resolution Task). Vertical error bars, ±1 SEM. A Green dashed line represents the chance-level choice rate (50% choice rate in two alternative forced choice). Mean BTS in the depth task at 120 cpd was significantly higher than chance-level (*t* = 5.45, df = 7, *p* < 0.01). Mean BTS in the depth task at 30 cpd was significantly lower than chance-level (*t* = -3.73, df = 7, *p* < 0.01) **(B)** Mean correlation coefficient between BTS and the image resolution in Depth and Resolution tasks (*n* = 8 each). The mean correlation coefficient between depth sensation and the image resolution was 0.90 (*n* = 8, *p* < 0.01, against zero). The mean correlation coefficient between resolution sensation and the image resolution was -0.01 (*n* = 8, *p* = 0.98, against zero).

The results show that participants’ depth sensation positively correlated with the stimulus resolution (a red line on **Figure 6A**, and the mean correlation coefficient between depth sensation and the image resolution was 0.90 ± 0.03 (*n* = 8, *p* < 0.01, against zero) on **Figure 6B**). On the other hands, in Resolution Task, there were no significant difference between the performance scores (a blue line) and the chance-level of choice rate (a green dashed line) (**Figure 6A**), and the mean correlation coefficient was -0.01 ± 0.33 (*n* = 8) (**Figure 6B**). It indicates that they were not able to discriminate the resolution difference. The results of the tumbling E visual acuity test show that there were no significant visual acuity differences between Depth and Resolution Task groups. Therefore, in the same fashion as Experiment 1 (Gabor patch), higher resolution image produces stronger depth sensation even without noticing the resolution difference.

### Comparison between Gabor Patch and Cylinder

To compare psychophysical results (correlation coefficient) in Gabor patch experiment with ones in Cylinder experiment, we conducted 2 × 2 ANOVA with two images (Gabor patch and Cylinder) and task conditions (Depth and Resolution tasks) as factors. As a result, the main effect of “Image” was not significant, on the other hand, the main effect of “Task condition” was significant (**Table 1**).

**Table 1:** Results of multifactor ANOVA with Image and Task condition.

	df	Mean square	*F-*value	*p*-value
Image	1	0.044	0.124	0.727
Task condition	1	4.707	13.245	0.001^∗∗^
Image × Task condition	1	0.291	0.819	0.372
			^∗^*p*<0.05	^∗∗^*p* < 01


*The main effect of Task condition was significant ($\eta_{p}^{2}$ = 0.41). This indicates that we obtained the same tendency from psychophysical results in both images, Gabor patch and Cylinder.*

These outcomes also demonstrate, in both Gabor patch and Cylinder experiments, higher resolution image enhances depth sensation even when participants did not detect the resolution difference.

## Discussion

In the present study, we investigated the relationship between spatial resolution of a visual image and depth sensation with Gabor patch and Cylinder image. Results in both experiments clearly showed that higher resolution images produce stronger depth sensation even without noticing the resolution difference. These results are in accordance with previous findings (Tsushima et al., 2014). Therefore, such concern in which participants did the Depth task by the aspect of high spatial frequency components at the edge line between Cylinder and the background did not notably appear to contribute to the previous findings. Instead our results suggest that image resolution is a general factor for depth perception for the following two reasons: first, Gabor patch is more appropriate stimulus for examining detection/discrimination ability for luminance-contrast difference/change than Cylinder image, because Gabor patch is much better fitted to the receptive field aspects of neurons in primary visual cortex (Judson and Palmer, 1987). Secondly, getting depth information from Gabor patch might be more fundamental visual function than the one from Cylinder image, because Depth task with Gabor patch asked participant to gain only depth information without any detailed object shape. In other words, depth information from Gabor patch was primarily formed from the simple luminance-contrast difference/change. This view might have some impact on the expression style of an image on the contemporary display.

Although we obtained the consistent psychophysical results in which higher resolution image makes stronger depth sensation, an important question still remains unanswered: why does higher resolution image facilitate our depth sensation? One of possible explanations is that the spatial frequency components of a visual stimulus manipulated by the display resolution crucially affects our depth perception. Previous studies proposed a model that low spatial frequency (LSF) components of a visual image provide the foundation for a visual cognition (Bar, 2003; Bar et al., 2006; Eger et al., 2007). The definition of visual cognition is different from the one of visual perception: cognition refers to the information process regulated by prior knowledge, memory, or learning, on the other hand, perception is the process of getting information from sensory organs (Gibson, 1979). In terms of this viewpoint, making the third dimensional information from the two dimensional image in this study is more of cognition than perception, because participants in Depth task made a decision based on prior knowledge in which luminance-contrast difference/change presented on the two dimensional image was useful as a depth cue. Therefore, if this is the case, lower resolution image produces less depth information, because it presumably has relatively larger amount of high spatial frequency components generated by such pixelated images (See the right image on **Figure 4B**). On the other hand, higher resolution image composed of relatively larger amount of LSF components (see the left image on **Figure 4B**) might more effectively provide depth information with our visual system (Bar, 2003; Bar et al., 2006; Eger et al., 2007). However, since the spatial frequency components of the visual images in this study were not well-controlled (See Supplementary Material), further studies must be needed to verify this hypothetical model.

At the same time, one of the intriguing points from the present results is that participants could not consciously access more primitive perceptual information (resolution) but higher cognitive information (depth information). This finding demonstrates the existences of consciously available and unavailable information in our visual system (Tong, 2003). Furthermore, even unattended and subliminal stimulus feature (e.g., resolution here) affects our visual perception (e.g., depth perception here) (He et al., 1996; Tsushima et al., 2006; Tsushima, 2014). In addition, since participants were aware of only higher cognitive information (depth information), our consciousness is mainly constituted through not bottom–up but top–down processing (Block, 1995, 2007). This might give us an important clue to answer one of the central questions about psychology, what is consciousness/unconsciousness?

The current study and the previous findings (Tsushima et al., 2014) have revealed that higher resolution image provides us with stronger depth sensation without recognizing the resolution difference, in a variety of images. Although the further studies should be conducted for fully understanding the mechanism, studying the interaction between new media technologies and our perceptual system shows us a new behavioral neuroscience phenomenon. That might give us a novel way in which we enjoy digital contents as well as a new insight of our neural system.

## Author Contributions

TM was involved in designing a research. YS and KK designed the study, and analyzed behavioral data. YT designed the study, collected, analyzed behavioral data, and wrote the paper. All authors discussed the results and commented on the manuscript.

## Conflict of Interest Statement

The authors declare that the research was conducted in the absence of any commercial or financial relationships that could be construed as a potential conflict of interest.
