# Supplementary material for: Undetectable Changes in Image Resolution of Luminance-Contrast Gradients Affect Depth Perception
Source: Front Psychol. 2016 Feb 23;7:242. doi: 10.3389/fpsyg.2016.00242 (PMC4763190; doi:10.3389/fpsyg.2016.00242)
Supplement: Supplementary file 1 [file Data_Sheet_1.PDF]

Supplementary Material:

“Undetectable changes in image resolution of luminance-contrast gradients affect depth perception.”

Yoshiaki Tsushima, Kazuteru Komine, Yasuhito Sawahata, and Toshiya Morita

Spatial frequency components of used images (Gabor patches)

Here, we show the spatial frequency components of Gabor patches as reference sources for better understanding a hypothetical model proposed in the main manuscript (Bar, 2003; Bar et al., 2006; Eger et al., 2007).

Calculation of power ratio between LSF and HSF components

First, we picked up a central area that might be crucially important for depth perception of luminance-contrast changes of Gabor patch used in the experiments (It indicates a pink square in Supplementary Figure A). Such image was transformed into a spatial frequency domain by applying two dimensional discrete cosine transform (2D-DCT), and represented by a square matrix in which each element corresponded to weight values for cosine basis functions with different spatial frequencies. We obtained the low and high spatial frequency (LSF and HSF) components of an image by computing squared sums of the spatial frequency components less than 7 cpd (double of spatial frequency of Gabor patches in the experiments), which corresponded to weight values within an upper left triangle of a matrix, and the farther high frequency elements (the outsides of the upper left triangle), respectively. Finally, the power ratio was calculated by dividing the computed LSF by the HSF + LSF components.

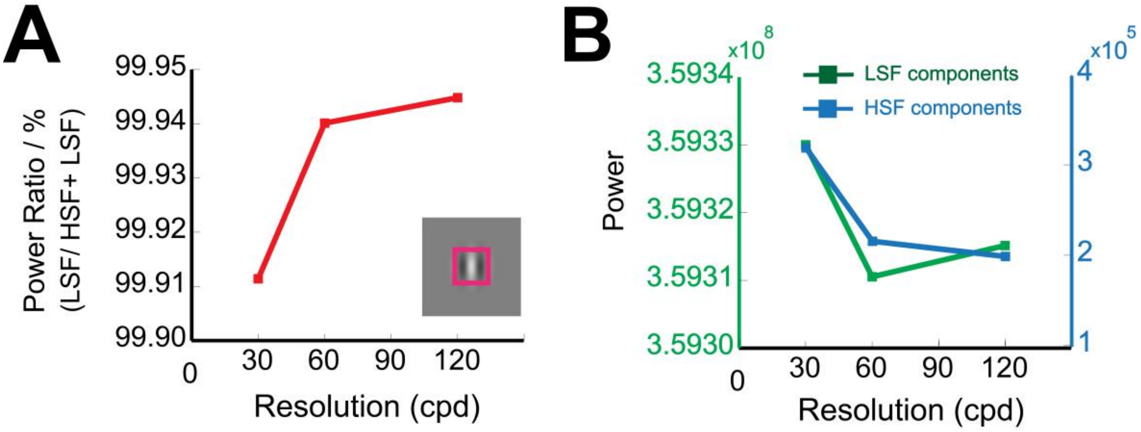

SUPPLEMENTARY FIGURE | Power ratios and powers of spatial frequency for Gabor patches

(A) Power ratio between LSF components / HSF + LSF components. **(B)** Powers of HSF and LSF components.

From these results, the power ratio (LSF components / HSF + LSF components) (Supplementary Figure A) might show some correlation with depth sensation in the Gabor patch experiment (Figure 5A in the main text). It partly provides an evidence for

validity of the hypothetical model in which LSF components of a visual image produce the foundation for visual cognition (Bar, 2003; Bar et al., 2006; Eger et al., 2007). However, as we mentioned in the main manuscript, HSF components and LSF components itself were not controlled well for testing this hypothesis. For example, the power of LSF components was not systematically changed with image resolution (Supplementary Figure B).
